# Two new species of Tipula (Vestiplex) from Southern China based on morphological and molecular data, with redescription of Tipula (Vestiplex) bicalcarata (Diptera, Tipulidae, Tipulinae)

**DOI:** 10.3897/zookeys.658.9738

**Published:** 2017-02-23

**Authors:** Qiu-Lei Men, Chen W. Young, Pavel Starkevich, Yong-Fu Yu, Xiao-Ping Lei

**Affiliations:** 1 School of Life Sciences, Province Key Laboratory of the Biodiversity Study and Ecology Conservation in Southwest Anhui; Research Center of Aquatic Organism, Conservation and Water Ecosystem Restoration in Anhui Province, Anqing Normal University, Anqing City 246011, Anhui, P. R. China; 2 Section of Invertebrate Zoology, Carnegie Museum of Natural History, 4400 Forbes Avenue, Pittsburgh, Pennsylvania 15213-4080, USA; 3 Nature Research Centre, Akademijos str. 2, LT-08412, Vilnius, Lithuania; 4 Administrative Bureau of Leigongshan National Nature Reserve, Leishan County 557199, Guizhou, P. R. China; 5 Administrative Bureau of Fanjingshan National Nature Reserve, Jiangkou County 554400, Guizhou, P. R. China

**Keywords:** China, COI, crane flies, Diptera, new species, sperm pump, Tipulidae, *Vestiplex*

## Abstract

Two new species of subgenus Tipula (Vestiplex) Bezzi, 1924, Tipula (Vestiplex) leigongshanensis Men & Young, **sp. n.** and Tipula (Vestiplex) maoershanensis Men & Young, **sp. n.** are described and illustrated. Tipula (Vestiplex) bicalcarata Savchenko, 1965 is redescribed and illustrated based on additional morphological characters. Partial mitochondrial cytochrome oxidase subunit I (COI) sequences of these three species are provided. Pairwise genetic distances among two new species and related species, Tipula (Vestiplex) bicalcarata, Tipula (Vestiplex) coxitalis Alexander, 1935, and Tipula (Vestiplex) sternotuberculata Alexander, 1935 range from 0.028 to 0.091 using Kimura-2-parameter model. Diagnostic features of the sperm pump for taxonomic use are discussed.

## Introduction

Tipula (Vestiplex) Bezzi, 1924 is a large subgenus in *Tipula* Linnaeus, 1758 with 170 species worldwide, distributed mainly in Oriental and Palaearctic regions (Oosterbroek 2016). Tipula (Vestiplex) was erected by Bezzi (1924) with the type species *Tipula cisalpina* Riedel, 1913 from the West Palaearctic region by original designation. China hosts 67 species of this subgenus, distributed mainly in the southern part of the country (Oosterbroek 2016). It is characterized by the following characters: antennae short to elongate, with flagellum very strongly incised in male; thorax with prescutum generally glabrous; tibial spur with formulation 1-2-2; squama naked; R_1+2_ entire, Rs one-half longer than m-cu; male hypopygium generally very sclerotized and blackened, with ninth tergite forming a saucer and produced into a pair of acute projections, gonocoxite generally elongated; female ovipositor with cerci heavily sclerotized and equipped with saw-like teeth on the lower margins, but smooth in some species; hypovalvae small, extending scarcely beyond the base of cerci (Alexander 1935a).

Many species of subgenus Tipula (Vestiplex) were originally placed in the subgenus Oreomyza Pokorny, 1887, which was subsequently treated as a synonym of the subgenus Tipula (Pterelachisus) Rondani, 1842. The subgeneric status of species in these two subgenera has always been a troublesome issue to crane fly researchers when identifying from morphological characters only. Therefore, molecular characters have become an important addition to morphological characters, and have been proven successful for separating and identifying insect species when applied to the following instances: fragmented specimens, closely related species with extremely similar morphology, cryptic species, dubious correspondence between larva and adult, or male and female (Hebert et al. 2004, Barcenas et al. 2005, Johanson 2007, Men and Qin 2011, Yang et al. 2012, Serjeant and Beebee 2013), as well as defining the taxonomic status of taxa (Lim et al. 2013, Reijnen et al. 2014).

Two new species of the subgenus Tipula (Vestiplex) were noticed among recently collected specimens from Guangxi Zhuang Autonomous Region and Guizhou Province in southern China. The present paper provides the descriptions and illustrations of the external morphology of the new species. The COI sequences of the new species are also provided in order to augment characteristic data. The COI sequence data were used to calculate the pairwise genetic distances among the new species and related species, to delineate and establish the two new species. And finally, the subgeneric position of new species is argued based on COI sequences of the new species and some known species of subgenera Tipula (Vestiplex) and Tipula (Pterelachisus). Tipula (Vestiplex) bicalcarata is redescribed and illustrated based on additional morphological characters. New distribution records for Tipula (Vestiplex) bicalcarata are provided. Diagnostic features and use of the sperm pump for taxonomy are discussed.

## Material and methods

### Taxonomic analysis

Photographs of the body parts of male adults were obtained using a SOIFXTZ-E stereomicroscope (SOIF, Shanghai, China). The hypopygium of each male was removed and macerated in 10% NaOH for one hour in a 50°C water bath, observed in glycerin and illustrated under a SOIFXTZ-E stereomicroscope (SOIF, Shanghai, China). The body length was measured from the vertex of head to the tip of hypopygium. All measurements were made in millimeters (mm) with the aid of a digital caliper. The angles between compressor apodemes and posterior immovable apodemes of the sperm pump were measured by ImageJ software. The terminology and methods of description and illustration followed that of Alexander and Byers (1981) and Frommer (1963). The type specimens are deposited in the animal specimen room, School of Life Sciences, Anqing Normal University, Anhui Province, P. R. China. Qiu-Lei Men and Chen W. Young were responsible for the taxonomic portion of this paper, thus are the authors of the new species.

### Molecular analysis

Genomic DNA was extracted from one leg of dry preserved specimen using Biomiga Insect gDNA Kit (Biomiga, USA). Genomic DNA of four type specimens of new species and two specimens of Tipula (Vestiplex) bicalcarata was extracted. The partial sequence of the mitochondrial COI gene was amplified using the universal primers for metazoan invertebrates, LCO1490 (5’-GGTCAACAAATCATAAAGATATTG-3’) and HCO2198 (5’-TAAACTTCAGGGTGACCAAAAAAT-3’) (Folmer et al. 1994). PCR amplifications were employed using a final volume of 20 μl containing 10 μl 2 × Pfu PCR MasterMix (Tiangen, Beijing, China), 0.75 μl each primer (10 μM), 1 μl DNA template and 7.5μl ddH_2_O. PCR amplification was employed with denaturation at 95 °C for 5 mins, followed by 45 cycles of 30 s at 95 °C for denaturation, 30 s at 50 °C for annealing and 1 min 30 s at 72 °C for extension, with a final extension at 72 °C for 10 mins. All PCR sets included a negative control reaction tube in which all reagents were included but the template DNA. After electrophoresis with 1% agarose gel, the target DNA was sent to Genescript Biotechnology Co., Ltd. (Nanjing, China) for sequencing. The partial COI sequences were aligned with CLUSTAL X (Thompson et al. 1997). The aligned sequences were processed by MEGA 6.0 (Tamura et al. 2013) for analyzing the DNA sequence compositions and calculating pairwise genetic distance based on the Kimura-2-parameter model (Kimura 1980). Partial COI sequences of new species and Tipula (Vestiplex) bicalcarata obtained in this study were submitted to GenBank with the following accession numbers: Tipula (Vestiplex) bicalcarata (KU844262), Tipula (Vestiplex) leigongshanensis sp. n. (KU844261) and Tipula (Vestiplex) maoershanensis sp. n. (KU844263). For revealing the subgeneric position of new species, a maximum likelihood tree was constructed using MEGA 6.0 with 1000 bootstraps (Tamura et al. 2013) based on COI sequences of the new species and known species of Tipula (Vestiplex) and Tipula (Pterelachisus), which were mainly downloaded from GenBank (accession numbers presented in Table 1).

**Table 1.** Accession numbers and sources of COI sequences of some known species in Tipula (Pterelachisus) and Tipula (Vestiplex).

**Species**	**Accession numbers**	**Sources**
Tipula (Pterelachisus) stenostyla	JQ912049	Pilipenko et al. 2012
Tipula (Pterelachisus) winthemi	JQ912057	Pilipenko et al. 2012
Tipula (Pterelachisus) jutlandica	JQ912035	Pilipenko et al. 2012
Tipula (Pterelachisus) octomaculata	JQ912044	Pilipenko et al. 2012
Tipula (Pterelachisus) submarmorata	JQ912050	Pilipenko et al. 2012
Tipula (Pterelachisus) pseudovariipennis	JQ912047	Pilipenko et al. 2012
Tipula (Pterelachisus) varipennis	JQ912054	Pilipenko et al. 2012
Tipula (Pterelachisus) mutila	JQ912042	Pilipenko et al. 2012
Tipula (Pterelachisus) wahlgreni	JQ912055	Pilipenko et al. 2012
Tipula (Pterelachisus) truncorum	JQ912051	Pilipenko et al. 2012
Tipula (Vestiplex) arctica	KU374459	Wirta et al. 2016
Tipula (Vestiplex) canadensis	KM571431	Barcoding Canada Data Release
Tipula (Vestiplex) bicalcarata	KU844262	New submission in present study
Tipula (Vestiplex) coxitalis	Not released	Provided by second author
*Tipula* (*Vestiplex) sternotuberculata*	Not released	Provided by second author

## Results

### COI sequences analysis

Sequences containing 623 base pairs were recovered for the studied species, which included 75 variable sites and 548 conserved sites. Variable sites are shown in Figure 38. Variable sites included 25 parsimony informative sites and 50 singleton sites. The pairwise genetic distances between the two new species and related species ranged from 0.028 to 0.091 based on Kimura-2-parameter model (Table 2).

**Table 2.** Pairwise genetic distances for COI gene sequences of species examined in the present study.

Species	*lei*	*ste-t*	*cox*	*bic*	*mao*	*win*	*wah*	*var*	*sub*	*ste-s*	*pse*	*oct*	*mut*	*jut*	*arc*	*can*
*ste-t*	0.086															
*cox*	0.042	0.084														
*bic*	0.059	0.088	0.057													
*mao*	0.047	0.091	0.040	0.028												
*win*	0.117	0.126	0.119	0.105	0.109											
*wah*	0.138	0.139	0.136	0.128	0.132	0.067										
*var*	0.126	0.132	0.124	0.118	0.118	0.057	0.077									
*sub*	0.138	0.141	0.134	0.130	0.132	0.062	0.089	0.034								
*ste-s*	0.132	0.128	0.132	0.111	0.118	0.038	0.084	0.079	0.072							
*pse*	0.138	0.134	0.130	0.130	0.130	0.069	0.085	0.019	0.041	0.088						
*oct*	0.134	0.134	0.136	0.121	0.119	0.054	0.086	0.080	0.084	0.064	0.082					
*mut*	0.134	0.134	0.140	0.128	0.138	0.075	0.028	0.085	0.089	0.082	0.094	0.087				
*jut*	0.128	0.132	0.124	0.117	0.117	0.039	0.074	0.077	0.081	0.054	0.077	0.066	0.081			
*arc*	0.132	0.136	0.128	0.128	0.132	0.137	0.138	0.142	0.148	0.140	0.148	0.154	0.144	0.150		
*can*	0.136	0.136	0.134	0.127	0.130	0.121	0.140	0.144	0.150	0.122	0.142	0.145	0.129	0.133	0.088	

Abbreviation: *lei*, Tipula (Vestiplex) leigongshanensis; *ste-t*, Tipula (Vestiplex) sternotuberculata; *cox*, Tipula (Vestiplex) coxitalis; *bic*, Tipula (Vestiplex) bicalcarata; *mao*, Tipula (Vestiplex) maoershanensis; *win*, Tipula (Pterelachisus) winthemi; *wah*, Tipula (Pterelachisus) wahlgreni; *var*, Tipula (Pterelachisus) varipennis; *sub*, Tipula (Pterelachisus) submarmorata; *ste-s*, Tipula (Pterelachisus) stenostyla; *pse*, Tipula (Pterelachisus) pseudovariipennis; *oct*, Tipula (Pterelachisus) octomaculata; *mut*, Tipula (Pterelachisus) mutila; *jut*, Tipula (Pterelachisus) jutlandica; *arc*, Tipula (Pterelachisus) arctica; *can*, Tipula (Pterelachisus) canadensis.

The maximum likelihood tree (Fig. 39) showed that the two new species clustered with species of Tipula (Vestiplex), suggesting their subgeneric position.

## Taxonomy

### Tipula (Vestiplex) bicalcarata

Taxon classificationAnimaliaDipteraTipulidae

Savchenko, 1965

#### Diagnosis.

Body generally reddish brown in color (Figs 1–6). Ninth tergite separated by a deeply V-shaped notch, lateral angles produced into an ear-like lobe (Fig. 8). Gonocoxite extended into a long arm, directed caudally, widest at base and narrowed to apex (Figs 6–7).

**Figures 1–13.**
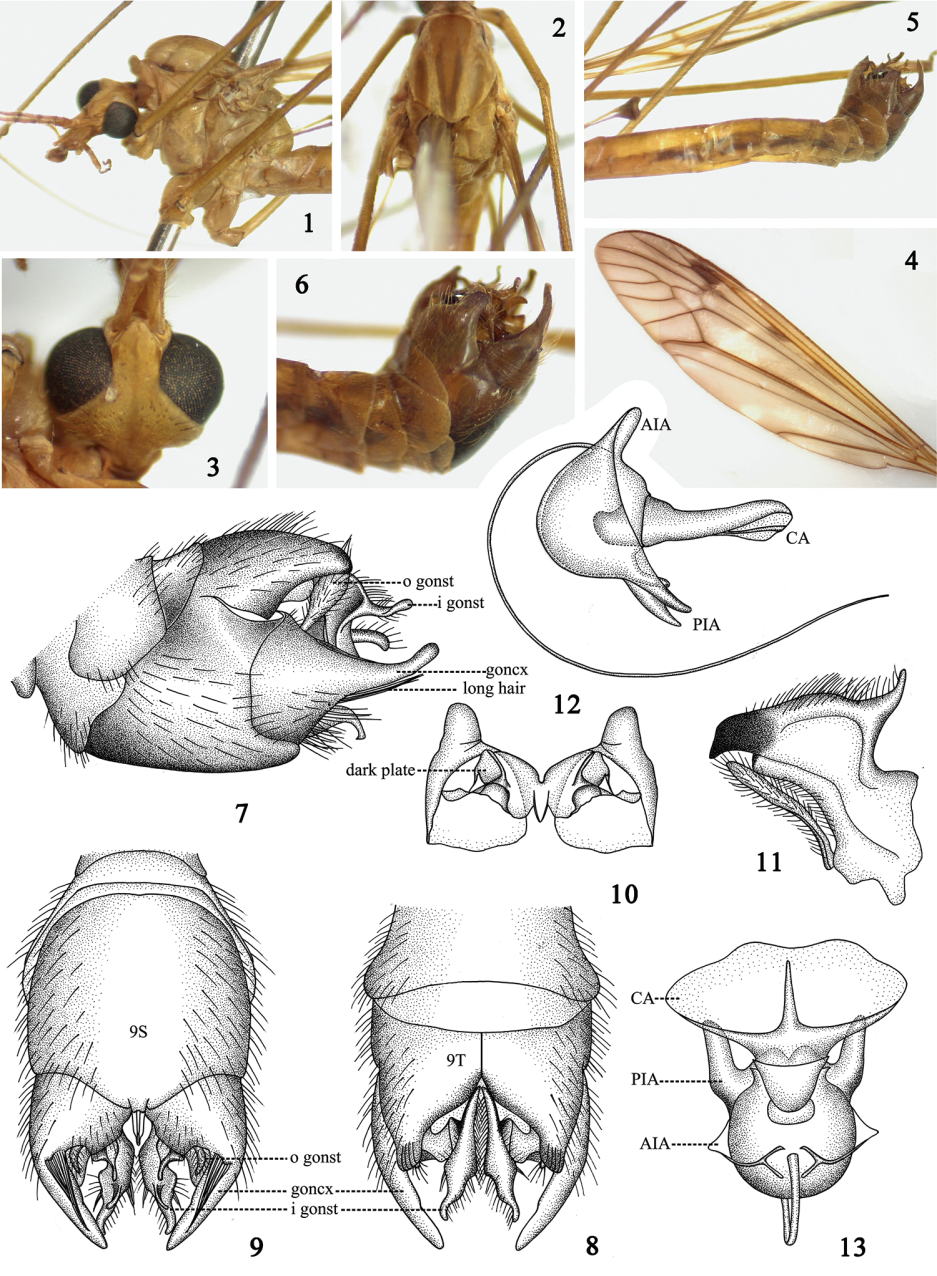
Tipula (Vestiplex) bicalcarata **1** thorax, lateral view **2** thorax, dorsal view **3** head, dorsal view **4** wing **5** abdomen and hypopygium, lateral view **6** hypopygium, lateral view **7** hypopygium, lateral view **8** hypopygium, dorsal view **9** hypopygium, ventral view **10** tergite nine, ventral view **11** inner gonostylus and outer gonostylus **12** sperm pump, lateral view **13** sperm pump, dorsal view. Abbreviation: AIA, anterior immovable apodeme; CA, compressor apodeme; goncx, gonocoxite; i gonst, inner gonostylus; o gonst, outer gonostylus; PIA, posterior immovable apodeme.

#### Redescription.

Male. Length: *Body*: 12.5–13.0 mm (excluding antenna, n = 5); *Wing*: 19.0–19.5 mm (n = 5); *Antenna*: 4.3–4.5 mm (n = 5).

*Head*. Reddish-brown except as noted. Vertex without marking (Fig. 3). Antenna reddish-brown, 13-segmented, bent backward extending to root of halteres; scape cylindrical, expanded apically; pedicel short, hemispherical; each flagellomere subequal in length, basal enlargement black with abundant black verticils, longest ones subequal to length of corresponding flagellomeres. Palpi with basal three segments reddish-brown, last one black.

*Thorax*. Generally reddish-brown except as noted. Pronotum light yellow laterally, gradually becoming reddish-brown, black medially (Figs 1–2). Prescutum with three light brown stripes, median one marginally suffused with brown at basal half, humeral angle with a black marking at lateral side (Figs 1–2). Scutum with two light brown markings (Fig. 2). Scutellum with dark median stripe. Postnotum wholly reddish-brown. Pleura entirely reddish brown (Fig. 1). Legs slender, coxae, trochanters and femora reddish-brown, tibiae and tarsi light brown. Halteres stem reddish-brown, knob darker. Wings reddish-brown, cells c and sc darker than ground color; stigma dark brown; Rs suffused with dark brown at origin point; discal cell transparent; some large hyaline areas at cells m and a (Fig. 4). Venation: R_1+2_ entire, discal cell narrow, elongated, petiole of cell m_1_ distinctly shorter than discal cell (Fig. 4).

*Abdomen*. Generally reddish brown except as noted. Abdominal tergites with brown lateral stripes (Fig. 5). Hypopygium brown (Fig. 6). Tergite nine with a deeply V-shaped notch, separated medially into two parts, produced into ear-like lobe in lateral angle, beneath it with a dark plate on ventral side (Figs 8, 10). Sternite nine broad, not fused with tergite nine (Figs 6–7, 9). Gonocoxite extended into a long arm, directed caudally, widest at base, tapering to apex, ventrally of gonocoxite with a central band of long black setae (Figs 5–9). Outer gonostylus elongated, thin, generally curled as a tube (Fig. 11). Inner gonostylus tapering to apex, terminating in a black beak, with horn-shaped process on its dorsal side (Fig. 11).

*Sperm pump*. Compressor apodeme fan-shaped with two rounded extensions marginally (Fig. 11), forming a 45° angle with posterior immovable apodeme (Fig. 12). Posterior immovable apodeme distinctly shorter than compressor apodeme, gradually narrowed to apex (Fig. 12). Anterior immovable apodeme short, gradually narrowed to apex (Fig. 12). Aedeagus tubular, almost 3.0 times longer than sperm pump, acute apically (Fig. 12).

#### Examined material.

3 males, Guizhou Province, Fanjingshan Mountain, 27°55'N, 108°38'E﻿, 12 June 2015, Guoxi Xue leg. 2 males, Guangxi Zhuang Autonomous Region, Dayaoshan Mountain, 24°08'N, 110°11'E, 14 May 2016.

#### Distribution.

China (Beibei, Chongqing; new distribution records in China: Fanjingshan Mountain, Guizhou Province; Dayaoshan Mountain, Guangxi Zhuang Autonomous Region, see Fig. 40).

### Tipula (Vestiplex) leigongshanensis

Taxon classificationAnimaliaDipteraTipulidae

Men & Young sp. n.

http://zoobank.org/46AEB6CF-724D-4965-A698-A3B43E822DC6

#### Diagnosis.

**Figures 14–25.**
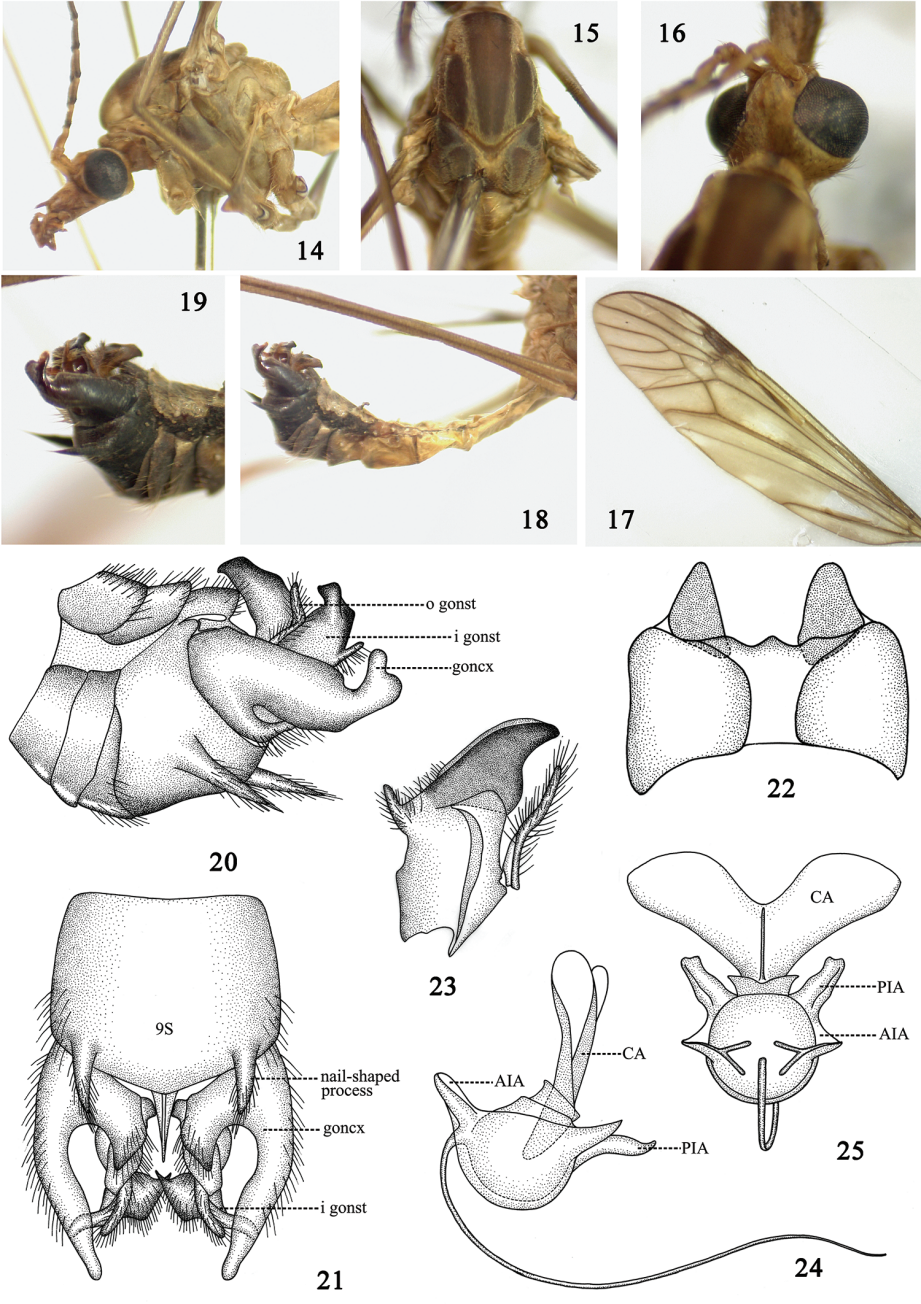
Tipula (Vestiplex) leigongshanensis, sp. n. **14** thorax, lateral view **15** thorax, dorsal view **16** head, dorsal view **17** wing **18** abdomen and hypopygium, lateral view **19** hypopygium, lateral view **20** hypopygium, lateral view **21** hypopygium, ventral view **22** tergite nine, dorsal view **23** inner gonostylus and outer gonostylus **24** sperm pump, lateral view **25** sperm pump, dorsal view. Abbreviation: AIA, anterior immovable apodeme; CA, compressor apodeme; goncx, gonocoxite; i gonst, inner gonostylus; o gonst, outer gonostylus; PIA, posterior immovable apodeme.

Body generally brown in coloration (Figs 14–19). Hypopygium expanded and black (Fig. 19). Sternite nine with a pair of nail-shaped processes (Figs 19–20). Gonocoxite produced into a bird-head-shaped lobe (Figs 19–20).

#### Description.

Male. Length: *Body*: 11.5–12.0 mm (excluding antenna, n = 4); *Wing*: 16.0–16.5 mm (n = 4); *Antenna*: 4.5–4.7 mm (n = 4).

*Head*. Generally reddish brown except as noted. Rostrum light brown with brownish nasus (Fig. 14). Antenna 13-segmented, bent backward extending to root of first abdominal segment; scape reddish-brown, cylindrical, expanded apically; pedicel reddish-brown, short; flagellum brown, flagellomere subequal in length, basal enlargement black with abundant black verticils, longest ones subequal to length of corresponding flagellomeres. Palpi entirely reddish brown. Vertex without marking (Fig. 16).

*Thorax*. Generally brown except as noted. Pronotum brown, becoming black medially. Prescutum with three brown stripes. Scutum with two dark brown markings (Fig. 15). Scutellum with a dark median stripe (Fig. 15). Postnotum entirely brown. Pleura reddish-brown, tinged with brown at anepimeron and anepisternum (Fig. 14). Legs slender, coxae and trochanters brown, femora brown with tip black, tibiae, and tarsi black. Halteres with stem brown, knob darker. Wings reddish-brown, cells c and sc darker than ground color; stigma dark brown; Rs suffused with dark brown at origin point; discal cell transparent; several large hyaline areas at cells r, m and a (Fig. 17). Venation: R_1+2_ entire, discal cell narrow, elongated, petiole of cell m_1_ distinctly shorter than discal cell (Fig. 17).

*Abdomen* with basal four segments brown, remaining segments generally darker, with black distinct median and lateral stripes, sternites entirely reddish-brown (Fig. 18). Hypopygium black (Fig. 19). Tergite nine separated medially into two parts, connected with membranous extension, hind margin of tergite nine forming W-shaped emargination (Fig. 22). Ventrad of tergite nine with two semi-triangular process (Fig. 22). Sternite nine broad, not fused with tergite nine, with a pair of nail-shaped processes arising from lateral sides, caudally directed, densely covered with long black setae (Figs 19–21). Gonocoxite produced into bird-head-shaped lobe, with small light-colored depression on base (Figs 19–20). Outer gonostylus elongated, thin, generally curled as a tube (Fig. 23). Inner gonostylus produced into black beak, with horn-shaped process on its dorsal side (Fig. 23).

*Sperm pump* with compressor apodeme V-shaped, forming a 55° angle with posterior immovable apodeme (Figs 24–25). Posterior immovable apodeme distinctly shorter than compressor apodeme, gradually narrowed to apex (Fig. 24). Anterior immovable apodeme short, gradually narrowed to apex (Fig. 24). Aedeagus tubular, almost 2.5 times longer than sperm pump, acute apically (Fig. 24).

#### Type material.

**Holotype** male. **CHINA**: Guizhou Province, Leigongshan Mountain, 26°21'N, 108°13'E, 2 June 2015, Guoxi Xue leg. **Paratype.** 1 male, same data as holotype. 2 males, Guizhou Province, Leigongshan Mountain, 26°21'N, 108°13'E, 14 May 2016, Qiulei Men leg.

#### Distribution.

China (Leigongshan Mountain, Guizhou Province, Fig. 40).

#### Remarks.

The new species is placed in subgenus Tipula (Vestiplex) because of its male hypopygium with elongated gonocoxite, which is also supported by the results of the molecular analysis (Fig. 39). The new species is mostly similar to Tipula (Vestiplex) sternotuberculata Alexander, 1935, from Taiwan, China, in the body color and the structure of hypopygium. Tipula (Vestiplex) leigongshanensis can be easily distinguished from the latter by the bird-headed distal end of gonocoxite (distal end of gonocoxite roundly expanded in Tipula (Vestiplex) sternotuberculata as figure 32 in Alexander, 1935b), and the nail-shaped process on sternite nine distinctly thinner than that of Tipula (Vestiplex) sternotuberculata. Moreover, pairwise genetic distance between these two species is 0.086 based on the Kimura-2-parameter model (the COI sequence of Tipula (Vestiplex) sternotuberculata is unpublished data, provided by the second author). Of 40 interspecific comparisons of genetic distance values among known species, 33 are equal to or lower than 0.086, which could suggest significant genetic variation between the new species and Tipula (Vestiplex) sternotuberculata (Table 2).

#### Etymology.

The specific epithet is a noun ‘*leigongshan*’ with Latin suffix ‘*ensis*’, referring to the type locality of the new species.

### Tipula (Vestiplex) maoershanensis

Taxon classificationAnimaliaDipteraTipulidae

Men & Young sp. n.

http://zoobank.org/9E2254C1-0F95-4CBA-994E-2649C913FF72

#### Diagnosis.

**Figures 26–37.**
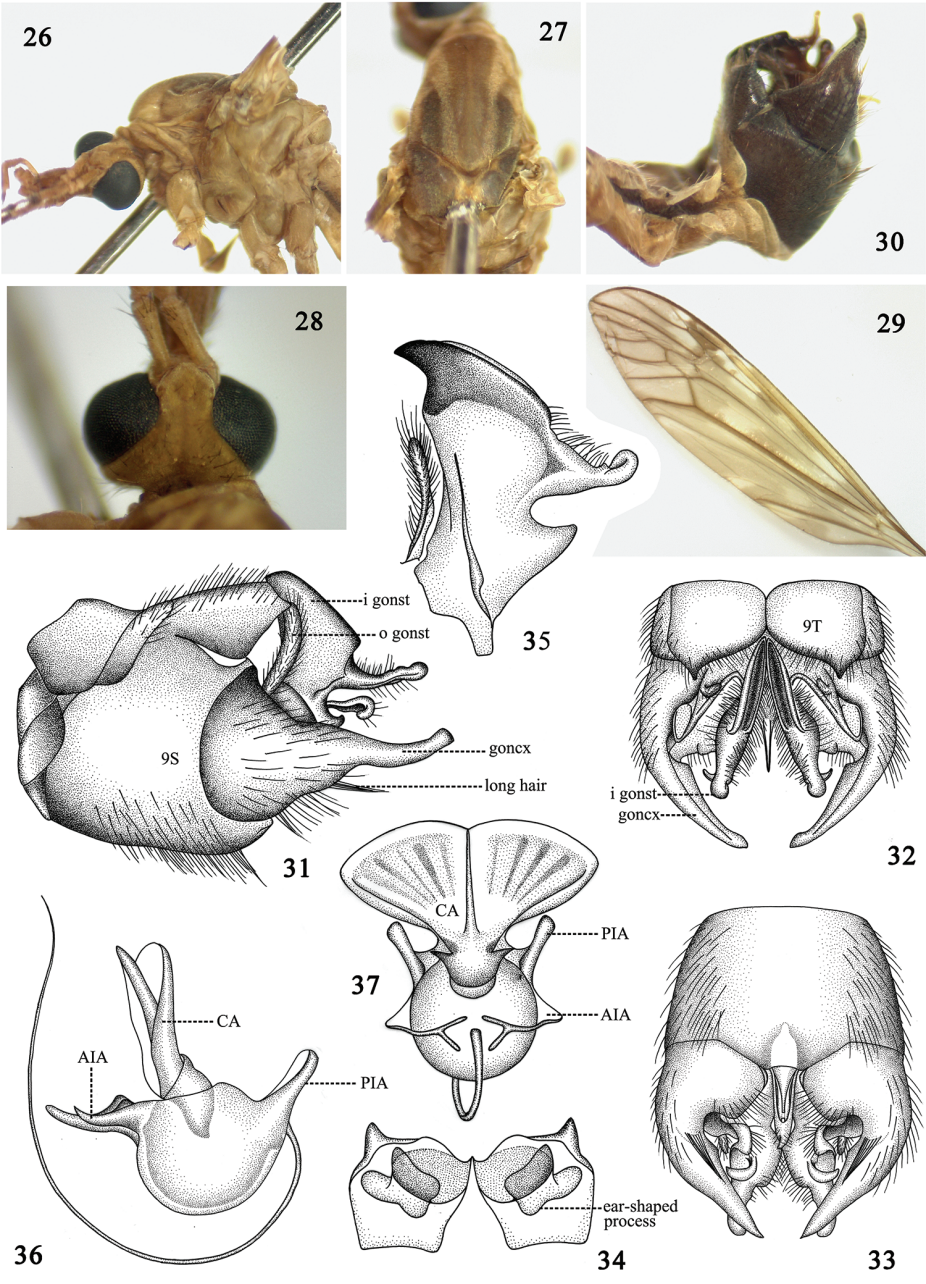
Tipula (Vestiplex) maoershanensis, sp. n. **26** thorax, lateral view **27** thorax, dorsal view **28** head, dorsal view **29** wing **30** hypopygium, lateral view **31** hypopygium, lateral view **32** hypopygium, dorsal view **33** hypopygium, ventral view **34** tergite nine, ventral view **35** inner gonostylus and outer gonostylus **36** sperm pump, lateral view **37** sperm pump, dorsal view. Abbreviation: AIA, anterior immovable apodeme; CA, compressor apodeme; goncx, gonocoxite; i gonst, inner gonostylus; o gonst, outer gonostylus; PIA, posterior immovable apodeme.

Whole body generally brownish in coloration (Figs 26–29). Prescutum with median stripe brighter than lateral stripes (Fig. 27). Hypopygium dark brown (Fig. 30). Gonocoxite produced into a long arm, basally broad and gradually narrowed to the apex (Figs 30–31).

#### Description.

Male. Length: *Body*: 12.0–12.3 mm (excluding antenna, n = 2); *Wing*: 16.2–16.5 mm (n = 2); *Antenna*: 3.5–3.7 mm (n = 2).

*Head*. Rostrum light brown with brownish nasus (Fig. 26). Antenna 13-segmented, bent backward extending to root of wing; scape reddish brown, cylindrical, expanded apically; pedicel reddish-brown, short; flagellum brown, flagellomere subequal in length, basal enlargement black with abundant black verticils, longest ones subequal to length of corresponding flagellomeres. Palpi entirely reddish brown. Vertex without marking (Fig. 28).

*Thorax*. Generally brown except as noted. Pronotum brown, changed to black medially. Prescutum with three brown stripes, median one darker than laterals (Fig. 27). Scutum with two dark brown markings (Fig. 27). Scutellum with dark median stripe. Postnotum wholly brown. Pleura entirely reddish-brown (Fig. 26). Legs slender, coxae and trochanters brown, femora brown with tip black, tibiae brown with tip black, tarsi black. Halteres with stem brown, knob darker. Wings reddish-brown, cells c and sc darker than ground color; stigma dark brown; Rs suffused with dark brown at origin point; discal cell transparent; several large hyaline areas at cells r, m and a (Fig. 29). Venation: R_1+2_ entire, discal cell narrow, elongated, petiole of cell m_1_ distinctly shorter than discal cell (Fig. 29).

*Abdomen*. Abdominal tergites reddish-brown with brown lateral stripes, sternites entirely reddish brown. Hypopygium dark brown (Fig. 30). Tergite nine entirely divided into two parts, produced into horn-shaped process in the lateral angle, ventral side of tergite nine with a pair of ear-shaped processes (Figs 32, 34). Gonocoxite extended into long arm, directed caudally, widened at base and narrowed to apex; ventrally, with central band of long black setae (Figs 30–31, 33). Outer gonostylus elongated, thin, generally curled as a tube (Fig. 35). Inner gonostylus produced into black beak, with a finger-shaped, rounded, expanded apically process on dorsal side.

*Sperm pump*. with compressor apodeme fan-shaped, shallowly emarginated in middle, deeper coloration in median region, suffused by several dark stripes, forming a 65° angle with posterior immovable apodeme (Fig. 37). Posterior immovable apodeme distinctly shorter than compressor apodeme, gradually narrowed to apex (Fig. 36). Anterior immovable apodeme short, gradually narrowed to apex (Fig. 36). Aedeagus tubular, almost 2.5 times longer than sperm pump, acute apically (Fig. 36).

#### Type material.

**Holotype** male. **CHINA**: Guangxi Zhuang Autonomous Region, Maoershan Mountain, 25°48'N, 110°25'E, 21 May 2015, Guoxi Xue leg. **Paratype.** 1 male, same data as holotype.

#### Distribution.

China (Maoershan Mountain, Guangxi Zhuang Autonomous Region, Fig. 40).

**Figure 38.**
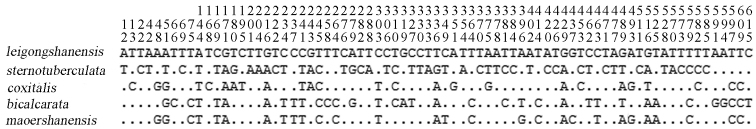
Variable sites from mitochondrial COI gene sequence alignment. Numbers arrayed vertically represent the positions of the nucleotides.

**Figure 39.**
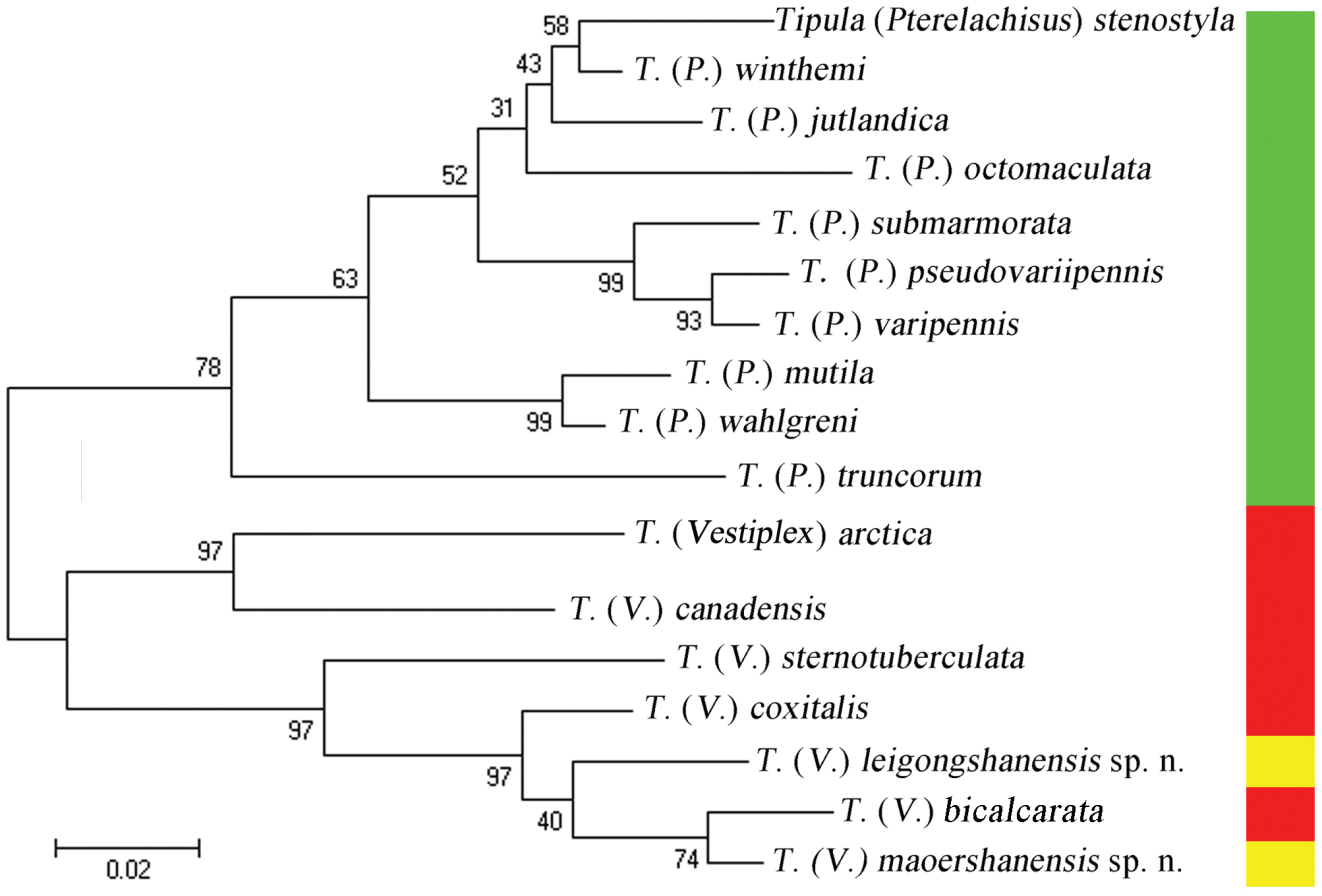
Maximum likelihood tree based on COI sequences of new species and some known species of Tipula (Vestiplex) and Tipula (Pterelachisus). Green region represents species of Tipula (Pterelachisus); red region represents species of Tipula (Vestiplex); yellow region represents new species.

**Figure 40.**
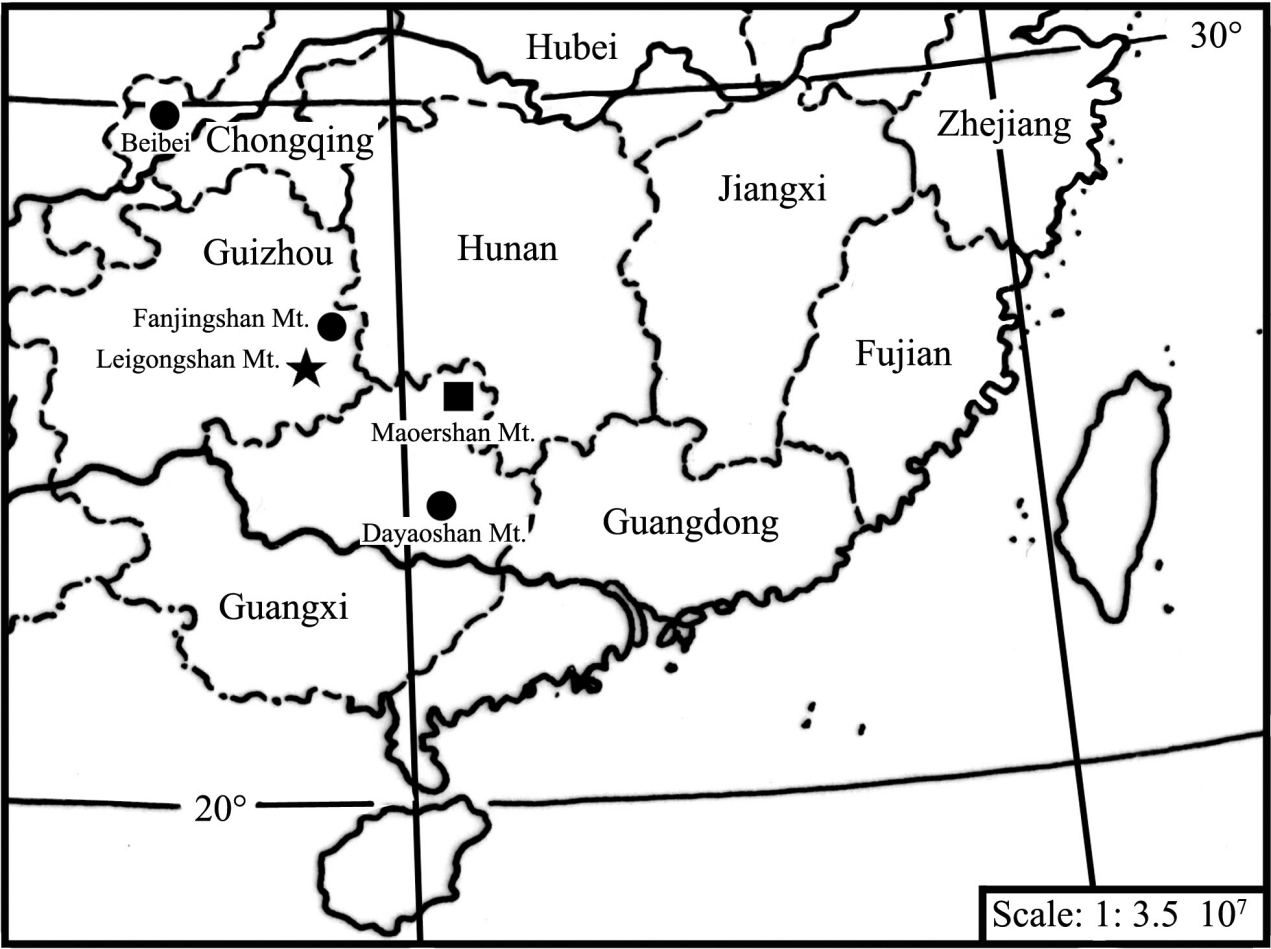
Geographic distribution of the species examined: Tipula (Vestiplex) bicalcarata (●), Tipula (Vestiplex) leigongshanensis sp. n. (★), Tipula (Vestiplex) maoershanensis sp. n. (■).

#### Remarks.

The new species is placed in subgenus Tipula (Vestiplex) because of its male hypopygium with elongated gonocoxite, which is also supported by the molecular analysis (Fig. 39). The new species is similar to Tipula (Vestiplex) coxitalis Alexander, 1935, in the body color and the structure of gonostylus, it can be easily distinguished from the latter by the gonocoxite not expanded distally (distal end of gonocoxite expanded in Tipula (Vestiplex) coxitalis figs 30, 31 in Alexander, 1935b), by the sternite without a band of long setae on each lateral sides (with such long hair in Tipula (Vestiplex) coxitalis fig. 30 in Alexander, 1935b), by the inner gonostylus with dorsal process distinctly larger than that of Tipula (Vestiplex) coxitalis. Pairwise genetic distance between Tipula (Vestiplex) maoershanensis sp. n. and Tipula (Vestiplex) coxitalis is 0.048 based on the Kimura-2-parameter model, suggesting distinct genetic variation between these two species. Six lower values of pairwise genetic distance were observed in comparisons to known species (Table 2), which could be taken as indirect evidence. The new species is also similar to Tipula (Vestiplex) bicalcarata by the body color and the structure of gonocoxite. It can be easily distinguished from the latter by the tergite nine produced into a horn-like process in lateral angle (the tergite nine produced into an ear-shaped process in Tipula (Vestiplex) bicalcarata, Fig. 8), the dorsal angle of inner gonostylus rounded and expanded apically (the dorsal angle of inner gonostylus gradually narrowed to apex in Tipula (Vestiplex) bicalcarata, Fig. 11). Pairwise genetic distance between these two species is 0.028 based on the Kimura-2-parameter model. Although the value is relatively low, it is equal to the comparison value of Tipula (Pterelachisus) wahlgreni and Tipula (Pterelachisus) mutila, while higher than the comparison value of Tipula (Pterelachisus) pseudovariipennis and Tipula (Pterelachisus) varipennis (0.019).

#### Etymology.

The specific epithet is a noun ‘*maoershan*’ with Latin suffix ‘*ensis*’, referring to the distribution of the new species.

## Sperm pump

The structures of sperm pumps in the two new species and Tipula (Vestiplex) bicalcarata showed substantial variation in shapes and colors, especially the shapes of the compressor apodemes, which suggests that the characters of sperm pumps can be used to distinguish closely related species (Table 3).

**Table 3.** Characters of sperm pump in three species of Tipula (Vestiplex).

Species	Compressor apodeme (CA)	Posterior immovable apodeme (PIA)	Anterior immovable apodeme (AIA)
*bicalcarata* (Figs 12, 13)	Fan-shaped, marginally with two rounded extensions. Generally reddish-brown.	Distinctly shorter than CA, narrow, acute apically, in a 45° angle with CA.	Small, inner lateral margins expended in lateral view, not connected apically.
*leigongshanensis* (Figs 24, 25)	Y-shaped. Generally reddish-brown.	Distinctly shorter than CA, narrow, acute apically, in a 55° angle with CA.	Gradually narrowed to apex, not connected apically. Small, inner lateral margins expended in lateral view.
*maoershanensis* (Figs 36, 37)	Fan-shaped, shallowly emarginated medially, deeper in coloration in median region, suffused by several dark stripes.	Distinctly shorter than CA, narrow, acute apically, in a 65° angle with CA.	Gradually narrowed to apex, not connected apically.

## Discussion

In most insect orders, males transfer their sperm to females by spermatophore (Khalifa 1949, Hünefeld and Beutel 2005). However, in most families of Diptera, sperm is transferred by means of semen pump or sperm pump, which also occurs in other members of Antliophora including Mecoptera and Siphonaptera (Hünefeld and Beutel 2005). The sperm pump of crane-flies shows rich morphological diversity in different taxa. sperm pumps have been successfully demonstrated to separate related species within the subgenus Tipula (Yamatotipula) and the genus *Nephrotoma*, based on the angle between the posterior immovable apodeme and the compressor apodeme, the length of posterior immovable apodeme, and the shape and color of compressor apodeme in previous studies (Men et al. 2015a, 2015b). Frommer (1963) divided the sperm pump of crane-flies into three types based on anatomical studies of male reproductive systems of tipuline crane-flies species from North American. In the present study, sperm pumps of new species belong to type III, which is the most common type characterized by the strongly bowed intromittent organ. After comparing the structures of sperm pumps in the three species examined in this present study, it was observed that they showed substantial variation in shapes of compressor apodemes and color, which indicates that the characters of sperm pumps are useful in distinguishing closely related species.

The pairwise genetic distance between Tipula (Vestiplex) maoershanensis sp. n. and Tipula (Vestiplex) bicalcarata was 0.028, the minimal value found when compared with the other three species, suggesting closer relationship and is in agreement with distinct morphological similarities between these two species. The distances between the two new species and the related species ranged from 0.028 to 0.091, within the range of 0.019 to 0.094, which covers all comparisons of known species inferred in the present study (Table 2). This may provide molecular evidence for the distinctiveness of these two new species.

China has a rich Tipuloidea fauna as indicated by the total numbers in the current catalogue (Oosterbroek 2016). Modern taxonomic studies on insects have been highly developed by integrating data derived from morphology, behavior, ecology, and geographic variation, which in turn is reinforced with complementary information from DNA sequences (Stoeckle 2003). Several published papers on Tipuloidea also show that the characterization of molecular data will likely contribute to our knowledge of the biodiversity and range extension of this group (Pilipenko et al. 2012, Goodman and Grady 2013, Wirta et al. 2016, Denes et al. 2016). We anticipate future intensive field collection and investigation would undoubtedly increase the species numbers and range extension of Tipula (Vestiplex) in China.

## Supplementary Material

XML Treatment for Tipula (Vestiplex) bicalcarata

XML Treatment for Tipula (Vestiplex) leigongshanensis

XML Treatment for Tipula (Vestiplex) maoershanensis
